# GABA_A_ Receptor Subunit α3 in Network Dynamics in the Medial Entorhinal Cortex

**DOI:** 10.3389/fnsys.2019.00010

**Published:** 2019-03-15

**Authors:** Nina Berggaard, Menno P. Witter, Johannes J. L. van der Want

**Affiliations:** ^1^Department of Clinical and Molecular Medicine, Faculty of Medicine and Health Sciences, Norwegian University of Science and Technology, Trondheim, Norway; ^2^Center for Computational Neuroscience, Egil and Pauline Braathen and Fred Kavli Center for Cortical Microcircuits, Kavli Institute for Systems Neuroscience, NTNU Norwegian University of Science and Technology, Trondheim, Norway

**Keywords:** GABA_A_ receptor subunit α3, medial entorhinal cortex, development, A-to-I editing, grid cells, Alzheimer’s disease

## Abstract

Layer II of the medial entorhinal cortex (MEC LII) contains the largest number of spatially modulated grid cells and is one of the first regions in the brain to express Alzheimer’s disease (AD)-related pathology. The most common principal cell type in MEC LII, reelin-expressing stellate cells, are grid cell candidates. Recently we found evidence that γ-aminobutyric acid (GABA)_A_ receptor subunits show a specific distribution in MEC LII, in which GABA_A_ α3 is selectively associated with reelin-positive neurons, with limited association with the other principal cell type, calbindin (CB)-positive pyramidal neurons. Furthermore, the expression of α3 subunit decreases in mice between P15 and P25, which coincides with the emergence of stable grid cell activity. It has been shown that the α3 subunit undergoes specific developmental changes and that it may exert pro-inflammatory actions if improperly regulated. In this review article, we evaluate the changing kinetics of α3-GABA_A_ receptors (GABA_A_Rs). during development in relation to α3-subunit expression pattern in MEC LII and conclude that α3 could be closely related to the stabilization of grid cell activity and theta oscillations. We further conclude that dysregulated α3 may be a driving factor in early AD pathology.

## Introduction

Most inhibitory signaling involves transmission of γ-aminobutyric acid (GABA) between neurons, and most of GABAergic signaling is mediated by ionotropic GABA_A_ receptors (GABA_A_Rs). These receptors are heteropentameric, consisting of five subunit proteins that together form a central chloride permeable pore. The subunit composition of GABA_A_Rs shows great variation throughout the brain. A total of 19 candidate subunit proteins can form a receptor: α1–6, β1–3, γ1–3, δ, ε, θ, π and ρ1–3 (Farrant and Nusser, 2005). The composition of a receptor often involves 2α, 2β and 1γ or 1δ subunits (Tretter et al., 1997; Nakamura et al., 2015). Different receptor compositions have different distributions on the postsynaptic cell membrane, along with different pharmacological properties and efficiency in mediating GABAergic neurotransmission.

One brain region, which shows a striking inhibition dominated local network, is layer II of the medial entorhinal cortex (MEC LII). With strong reciprocal connections to the hippocampus, the MEC is a major hub for the generation of an internal representation of space (Hafting et al., 2005; Sargolini et al., 2006; Solstad et al., 2008; Kropff et al., 2015). Principal cells of MEC LII can be classified into stellate and pyramidal cells, which largely express reelin (RE) and calbindin (CB), respectively, although intermediate cell types have also been described (Fuchs et al., 2015; Witter et al., 2017). MEC LII principal cells play essential roles in brain function. First, it has recently been found that RE+ cell activity drives the maturation of the entire entorhinal-hippocampal circuit (Donato et al., 2017). Moreover, both RE+ and CB+ cells encompass spatially modulated grid cells, which have a hexagonally arranged activity pattern spanning the explored environment (Domnisoru et al., 2013; Schmidt-Hieber and Häusser, 2013; Tang et al., 2014; Sun et al., 2015; Rowland et al., 2018). Most MEC LII principal cells are connected through fast-spiking perisomatic GABAergic parvalbumin-expressing (PV+) interneurons (Couey et al., 2013; Fuchs et al., 2015), which have been shown to be crucial for the emergence of grid cell activity (Buetfering et al., 2014; Miao et al., 2017). However, until recently, cell type-specific expression of GABA_A_R subunits has not been reported.

**Figure 1:**
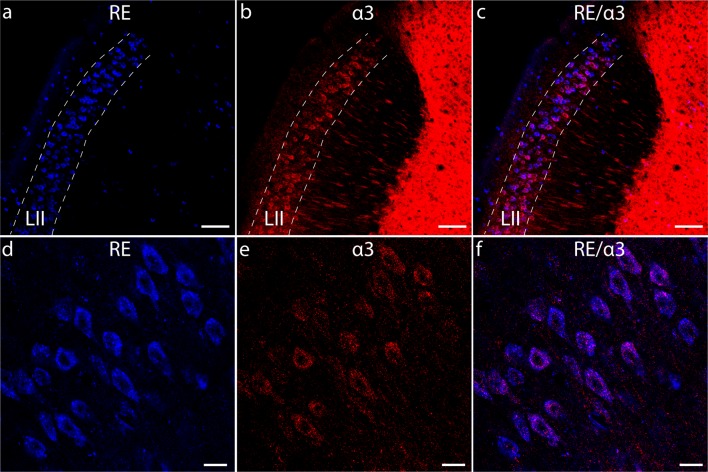
RE+ cells of layer II of the medial entorhinal cortex (MEC) LII express the GABA_A_R α3 subunit. **(A–C)** Overview of the dorsal portion of the adult mouse MEC from a sagittal section, stained for RE **(A)** and α3 **(B)**. Panel **(C)** shows an overlay of **(A,B)**. **(D–F)** High magnification images of RE **(D)** and α3 **(E)** immunoreactive cells in dorsal MEC LII. Panel **(F)** shows an overlay of **(D,E)**. Note the strong association between RE and α3 immunoreactivity. Dashed lines delineate LII. Scale bars: **(A–C)**, 100 μm; ** (D–F)**, 20 μm.

Previously we discovered that RE+ cells rather prominently express the GABA_A_R subunit α3 (α3-GABA_A_Rs; Figure 1; Berggaard et al., 2018b). Studies have shown that α3 may play important roles in neuronal development (Ohlson et al., 2007; Daniel et al., 2011), as well as in regulating anxiety and stress (Dias et al., 2005). Furthermore, emerging evidence suggests dysregulated activity by α3-GABA_A_Rs causes the subunit to become pro-inflammatory and to play important roles in the onset and progression of pathologies such as cancers and colon inflammation (Gumireddy et al., 2016; Liu et al., 2016; Long et al., 2017; Seifi et al., 2018). It is therefore possible the α3 subunit is involved in various processes in MEC LII besides mediating inhibitory neurotransmission.

In this review article, we will highlight the known involvement of α3 in physiological and pathological processes. We will further discuss the possible implications of the strong presence of α3 in RE+ cells of MEC LII, focusing particularly on early postnatal development, kinetics of GABAergic inhibition, and the possible role of this subunit in Alzheimer’s disease (AD). We will hereafter refer to the subunit protein and RNA as α3 and Gabra3, respectively.

### Kinetics of GABA_A_R Mediated Inhibition in MEC LII

In the mouse MEC LII, we found RE+ and CB+ principal cells to express distinct GABA_A_R subunits during a period implicated in grid cell maturation. Both cell types showed weak and strong expression of α1 and γ2, respectively, and no somato-dendritic expression of α2 and α4. The subunits α3 and α5 were instead largely specific to RE+ and CB+ cells, respectively, apart from a subset of CB+ cells expressing α3 (Berggaard et al., 2018b). Since the location and subunit composition of receptors on the postsynaptic cell membrane are important for determining the effect of inhibition, it is likely that GABAergic inhibition of the two cell types has different effects. GABA_A_Rs containing α1 and/or α3 in combination with γ2 subunits are predominantly located in the synapse where they mediate phasic inhibition in response to GABA release from the presynaptic terminal. In contrast, α5-GABA_A_Rs are expressed in extrasynaptic spaces where they mediate tonic inhibition (Brünig et al., 2002; Farrant and Nusser, 2005).

How fast GABA_A_Rs bind to, and dissociate from, GABA, is an important influencer of for instance oscillation frequency. Although α3-GABA_A_Rs predominantly mediate phasic inhibition, their activation and deactivation kinetics are in general slower than of α1-GABA_A_Rs. The activation kinetics of a receptor partly depends on its sensitivity to GABA. This can be found by measuring the concentration of GABA needed to elicit the half-maximal response (EC_50_; Farrant and Nusser, 2005). When comparing the EC_50_ values of receptors containing the αxβ3γ2 combination, it was found that α3β3γ2, and in one study also α2β3γ2, exhibit the highest EC_50_ values, and thereby lowest sensitivity to GABA compared to all other α subunits (Böhme et al., 2004; Mortensen et al., 2011). α3 subunits may, however, also exist in combination with ε and/or θ subunits, and it has been found that receptors with the α3β1ε combination are nearly 100-fold more sensitive to GABA compared to α3β1γ2 (Ranna et al., 2006). Furthermore, while open, the maximum GABA currents obtained with α3-GABA_A_Rs are larger than those obtained with α1- and α5-GABA_A_Rs (Mortensen et al., 2011; Table 1).

**Table 1:** GABA potency and maximal currents of various GABA_A_R subunit compositions.

Subunit composition	EC_50_ (μM)			GABA max currents (pA)
	Böhme et al. (2004)	Mortensen et al. (2011)	Ranna et al. (2006)	Mortensen et al. (2011)
α1β3γ2	2.9 ± 0.1	2.1		3,367 ± 662
α2β3γ2	5.2 ± 0.2	13.4		3,056 ± 435
α3β3γ2	48 ± 2	12.5		3,776 ± 305
α4β3γ2	7.6 ± 0.3	2.1		2,574 ± 292
α5β3γ2	11.6 ± 0.5	1.4		2,642 ± 938
α6β3γ2	1.0 ± 0.03	0.17		2,446 ± 445
α3β1ε			2.3 ± 0.5	
α3β1			55 ± 6	
α3β1θ			81 ± 18	
α3β1γ2			200 ± 38	

*EC_50_ is the concentration of GABA required to induce 50% of the maximal response. GABA maximal currents were measured at saturating concentrations of GABA. All values are mean ± SEM, except EC_50_ data from Mortensen et al. (2011), which are mean values*.

The deactivation kinetics are also much slower for α3-GABA_A_Rs compared to α1-GABA_A_Rs (Table 2), and are associated with slow desensitization rates of α3-GABA_A_Rs (Barberis et al., 2007; Mortensen et al., 2011; Eyre et al., 2012). This is in contrast to the extrasynaptic α5-GABA_A_Rs, which exhibit faster desensitization, but slower deactivation, such that the strength of GABAergic transmission decreases rapidly but lasts longer than in case of α3-GABA_A_Rs (Mortensen et al., 2011).

**Table 2:** Weighted decay time constants of miniature postsynaptic currents of α1- and α3-GABA_A_Rs.

Subunit composition	τ_W_ (ms)	
	Eyre et al. (2012)	Barberis et al. (2007)
α1xx	4–6	
α3xx	28	
α1β2γ2		52.5 ± 2.9
α3β2γ2		185.3 ± 30.1

Our previous study focused on the presence of α3 and not that of ε and θ subunits in MEC LII, thus it is unknown whether α3 combines with these subunits in RE+ cells. However, the strong expression of γ2 in RE+ cells (Berggaard et al., 2018b) renders it likely that at least a subset of α3-GABA_A_Rs in RE+ cells contain γ2. Although the sensitivity of these receptors to GABA is low, the high concentration of GABA following presynaptic release would still allow for fast activation, albeit slightly slower than for α1-GABA_A_Rs. Once α3-GABA_A_Rs are activated, their responsiveness to GABA remains high for a prolonged time until the receptors deactivate rather sharply. One of the key features of RE+ cells, apart from low input resistance and sag potential, includes high-frequency burst firing at the beginning of a spike train (Canto and Witter, 2012; Couey et al., 2013). Based on these observations, one can predict that the strong and prolonged inhibitory currents, followed by rather sharp deactivation of α3-GABA_A_Rs on the postsynaptic membrane, are among the factors that enable RE+ cells to enter into bursting mode.

### α3 Subunit in Development

Throughout development, there are alterations in subunit composition, distribution and kinetics of GABA_A_Rs. Laurie et al. (1992) applied *in situ* hybridization to demonstrate age-related changes in expression of 13 GABA_A_R subunits. They report changes in expression pattern of each individual GABA_A_R subunit mRNA during early development that coincide with the shift in GABA’s role from excitatory, neurotrophic factor to an inhibitory transmitter. During embryonic and early postnatal period of the rat, α3 and α2 subunits are the most widespread among the α subunits in the brain, followed by the α5 subunit. Moreover, α3 displays the highest mRNA levels among α subunits in the neocortex until postnatal day P6–12, after which it is largely replaced by the α1 subunit and restricted to the deeper cortical layers (Laurie et al., 1992; Wisden et al., 1992).

Simultaneous to a decrease in α3 protein expression during development, an increasing amount of Gabra3, its RNA counterpart, undergoes so-called adenosine-to-inosine (A-to-I) editing (Ohlson et al., 2007). A-to-I editing is catalyzed by Adenosine Deaminases that act on RNA (ADAR) enzymes and is a type of post-transcriptional processing of double-stranded RNA especially common in the human brain (Bass, 2002). Ever since A-to-I editing was discovered, tens of thousands of editing sites have been revealed. In addition, knowledge about the important roles of A-to-I editing in brain development and the involvement of improperly or unedited RNA in brain diseases has increased (Khermesh et al., 2016; Bajad et al., 2017). During editing of Gabra3, an isoleucine molecule is recoded into methionine by ADAR1 and ADAR2 of the ADAR family, at a highly evolutionary conserved genomic region (Ohlson et al., 2007; Daniel et al., 2011). The percentage of A-to-I edited Gabra3 displays a gradual increase from approximately 4%–7% at E15 to 53%–54% and 90%–93% at P2 and P7, respectively, which persists into adulthood (Rula et al., 2008; Wahlstedt et al., 2009; Ensterö et al., 2010). Slightly lower levels of edited Gabra3 at P7 (78%) with a subsequent increase to 92% at P21 were also observed (Daniel et al., 2011). In contrast, total levels of Gabra3 mRNA showed a sharp increase between mouse embryonic day E15 to E19, followed by a gradual decrease between postnatal day P7 and adulthood. High levels of edited Gabra3 were observed in all brain areas investigated, apart from the hippocampus, where only about 70% of Gabra3 was edited in the adult (Rula et al., 2008). In addition, the striatum has been found to have significantly lower levels of edited Gabra3 compared to the cortex (O’Neil et al., 2017).

A possible role of α3 is to maintain synapses. In a study on mice exhibiting targeted deletion of the α3 subunit, the GABAergic synapses in the reticular nucleus of thalamus were fewer and larger compared to wild type mice (Studer et al., 2006).

### α3 Subunit and GABA-Shift

There are indications that A-to-I editing of Gabra3 could facilitate the transition of GABA from excitatory to inhibitory. In the immature brain, GABAergic synapses mature prior to glutamatergic synapses (Tyzio et al., 1999; Khazipov et al., 2001; Ben-Ari et al., 2007). However, due to high intracellular concentration of Cl^−^, GABAergic activation of GABA_A_Rs at this stage results in an excitatory effect through an efflux of Cl^−^ ions. The shift results from an increase in the expression of potassium chloride channels (KCCs). During development there is an increase in expression of Cl^−^ extruding K^+^/Cl^−^ co-transporter KCC2, which causes reduced levels of intracellular Cl^−^ such that GABA_A_R-mediated signaling becomes hyperpolarizing (Rivera et al., 1999). This “GABA switch” occurs at slightly different time points in different brain regions and is dependent on GABAergic activation of GABA_A_Rs, as perturbed activation of GABA_A_Rs directly affects the mRNA levels of KCC2 as well as the timing of the switch (Ganguly et al., 2001; Leitch et al., 2005). In the chick retina, increased levels of A-to-I edited Gabra3 were found to go hand in hand with an increased expression of KCC2, which could imply that A-to-I editing of Gabra3 is an important component for the GABA switch (Ring et al., 2010).

Furthermore, editing of Gabra3 changes the kinetics of α3-GABA_A_R mediated inhibition. Electrophysiological recordings in human embryonic kidney cells have revealed that non-edited α3, in combination with β3 and γ2, have an average EC_50_ value of nearly half of that of edited α3, implying that the sensitivity to GABA is much higher for isoleucine-containing subunits compared to the ones containing methionine. Furthermore, the decay rate was found to be slower for unedited α3 compared to edited α3, suggesting that GABAergic transmission through α3-GABA_A_Rs is more effective in immature brain compared to the adult. The editing position in Gabra3 is in the third transmembrane region, which is a region known for regulating trafficking of α subunits of GABA_A_Rs. Thus, the expression of α3-GABA_A_Rs is directly affected by the isoleucine to methionine change. Confirmation of this notion was obtained in a study which showed that edited α3 displayed a 60% reduction in cell surface expression and approximately 40% reduction in total protein levels compared to non-edited α3, irrespective of subunit composition (Nimmich et al., 2009; Daniel et al., 2011).

### α3 Subunit: Possible Role in RE+ and Grid Cell Development?

In MEC LII, RE+ cells drive the development of the entorhinal-hippocampal circuit, including CB+ and PV+ cells in MEC LII. It was found that the maturation of RE+ cells is independent of any input from excitatory neurons, and instead depends on RE+ cell birth date, which suggests cell autonomous pathways being responsible for their maturation (Donato et al., 2017). The effect of input from GABAergic terminals on RE+ cells was, however, not investigated. This could be of interest considering these synapses are established prior to glutamatergic synapses. Since RE+ and CB+ cells largely express α3- and α5-GABA_A_Rs, respectively (Berggaard et al., 2018b), the cell type-specific instructive signal from GABAergic interneurons might promote development of RE+ cells following a dorsoventral gradient. Interestingly, in the early postnatal period of the rat, mRNA levels of most GABA_A_R subunits in the EC are higher than in the neighboring cortex (Laurie et al., 1992), which could imply increased GABAergic activity in this region compared to the surround. Moreover, ultrastructural investigations on the development of PV+ terminal input on rat MEC LII cell somata revealed that, at P10, close to all somata in the dorsal portion form synaptic contacts with PV+ terminals. Somata in the ventral portion, however, showed significantly less PV+ terminal apposition (Berggaard et al., 2018a).

In rodents, the emergence and stabilization of grid cell activity has been reported to happen after eye opening, from approximately P20, with the number of grid cells reaching adult levels around P22 (Wills et al., 2010, 2012; Tan et al., 2017). During this time, we noticed a significant decrease in both protein and mRNA levels of the α3 subunit, whereas the levels of other subunits measured were unchanged (Berggaard et al., 2018b). This decrease occurs in the same time frame with data on the change of Gabra3 levels in the brain during development, and may therefore serve a more widespread function, such as decreased α3/α1 ratio and consequently more rapid GABAergic transmission. However, since decreasing levels of Gabra3 have been directly associated with A-to-I editing of the subunit, the decrease seen in α3 levels between P15 and P25 may reflect α3-GABA_A_Rs in RE+ and a subset of CB+ cells undergoing A-to-I editing. This could be important, since at least a subset of grid cells likely contain α3-GABA_A_Rs. In case α3-GABA_A_Rs in immature grid cells undergo editing during the period that grid cell activity emerges, this would cause the kinetics of GABAergic inhibition in these cells to alter. A possible outcome of such an event is that the new kinetics of GABAergic transmission allow grid cell activity to stabilize.

### α3 Subunit Is Regulated by Sex Hormones

While most of the GABA_A_R receptor subunit genes appear in small clusters on autosomes, the α3, ε and θ subunits are the only known subunit genes that are positioned on the X chromosome. More specifically, they are located in close approximation to each other at the Xq28 position in humans, which is a candidate region for X-linked disorders such as early onset parkinsonism (Bell et al., 1989; Garret et al., 1997; Korpi et al., 2002; Kolb-Kokocinski et al., 2006). They might regulate the turnover of noradrenaline, dopamine and 5-HT (McKernan and Whiting, 1996).

Emerging evidence suggests that ovarian hormones regulate Gabra3. In a study on gonadotropin-releasing hormone neurons, which regulate reproduction, there were among several genes increased levels of Gabra3 mRNA in proestrous compared to metestrous female mice. This was in contrast to Gabra1-2 and Gabra5 mRNA levels, which were unchanged between the two groups (Vastagh et al., 2016). In addition, mRNA levels of Gabra3 were higher in male mice compared to metestrous female mice (Vastagh et al., 2015). In the dorsal raphe nucleus of female rhesus monkeys, there was a significant increase in Gabra3 RNA upon treatment with a combination of progesterone and estradiol, but not after estradiol treatment alone. Since progesterone and estradiol treatment also gave a reduction in RNA of JNK-1 and kynurenine 3-hydroxylase, which are pro-apoptotic and generate neurotoxic quinolones, this could imply that an upregulation of Gabra3 is neuroprotective in the serotonergic population (Reddy and Bethea, 2005). In addition, MEC LII is closely regulated by treatment with ovarian hormones, as shown in a study on ovariectomized rats with unilateral lesion of the perforant path. This ultimately causes ipsilateral degeneration of the RE+ cell population of EC LII. Here it was found that treatment with a combination of estradiol + discontinuous application of progesterone significantly improved neuronal survival and neurite outgrowth compared to estradiol + continuous progesterone administration or estradiol alone (Barron et al., 2015). The mechanisms underlying this neuroprotection could therefore in part be due to treatment-induced increased levels of Gabra3 in RE+ cells.

### α3-Subunit: Possible Role in Alzheimer’s Disease?

While the importance of A-to-I editing of Gabra3 is still being investigated, it becomes increasingly clear that a failure of proper A-to-I editing can cause various diseases (for review see Bajad et al., 2017). For example, studies on different types of cancers, including breast, pancreatic and lung, have implicated Gabra3 in the disease progression. Specifically, Gabra3 was found to promote cancer cell invasion and migration by activating various inflammatory pathways such as AKT/mTOR and JNK (Gumireddy et al., 2016; Liu et al., 2016; Long et al., 2017). In one of the studies, it was discovered that only the unedited form of Gabra3 had metastatic properties, while A-to-I edited Gabra3 was found to suppress cancer progression (Gumireddy et al., 2016). This could imply that dysregulated A-to-I editing of Gabra3 in the brain may also be pathological. With respect to MEC LII, there is a close link to AD, since it is one of the first regions in the brain to exhibit pathological changes associated with the neurodegenerative disease (Braak and Braak, 1991; Kobro-Flatmoen et al., 2016). AD has previously been associated with a reduction in A-to-I editing (Khermesh et al., 2016). In the frontal cortex of humans aged 22–102 years who had mostly died of heart failure, nearly all subjects had editing levels of Gabra3 of at least 90%, apart from two subjects who had died of skin cancer and hypoxia ischemia and whose editing levels were only 60% (Nicholas et al., 2010). Overall, these results suggest Gabra3 remains edited throughout life in the healthy brain, and that high levels of unedited Gabra3 are most likely pathological. It is therefore possible that the ratio of edited to unedited Gabra3 is lower in MEC LII of AD patients. In this regard, it is worth noting that many of the cell types which show increased vulnerability to AD pathology express α3, including strong expression on cholinergic, noradrenergic, dopaminergic and serotonergic systems (Gao et al., 1995; Rodríguez-Pallares et al., 2001; Corteen et al., 2015). Furthermore, early AD is associated with decreased power and frequency of gamma oscillations (Klein et al., 2016), which could be related to a change in activity of α3-GABA_A_Rs. The regulation of Gabra3 by ovarian hormones may thus be of relevance for AD, considering the typical AD patient is a postmenopausal woman.

## Conclusion

The GABA_A_R α3 subunit is strongly expressed in MEC LII, predominantly in RE+ stellate cells. In this review, we have discussed some potential consequences that the distribution of α3-GABA_A_Rs may have in refining GABAergic activity and strength in specific neurons and synaptic circuits in navigation. The α3 subunit undergoes an isoleucine-to-methionine change during early postnatal development, which significantly alters the kinetics of α3-GABA_A_R mediated inhibition. This editing event may coincide with, and be important for, the development of MEC LII cells, such as grid cells. Theta oscillation activity of grid cells matures during early developmental stages and shows a similar temporal pattern for the α3 subunit. Furthermore, the fact that α3 is regulated by ovarian hormones, and that a failure to properly edit α3 is likely pathological, could be a factor in AD.

## Data Availability

The datasets for this manuscript are not publicly available because the data are available upon request. Requests to access the datasets should be directed to johannes.want@ntnu.no.

## Author Contributions

NB, MW and JW wrote the article.

## Conflict of Interest Statement

The authors declare that the research was conducted in the absence of any commercial or financial relationships that could be construed as a potential conflict of interest.
